# Graphene nanowalls formation investigated by Electron Energy Loss Spectroscopy

**DOI:** 10.1038/s41598-023-51106-z

**Published:** 2024-01-18

**Authors:** Badri Vishal, Abdeldjalil Reguig, Mohammed Bahabri, Pedro M. F. J. Costa

**Affiliations:** Physical Science and Engineering Division, King Abdullah University of Science and Technology (KAUST), Thuwal, 23955-6900 Jeddah, Saudi Arabia

**Keywords:** Materials for energy and catalysis, Nanoscale materials

## Abstract

The properties of layered materials are significantly dependent on their lattice orientations. Thus, the growth of graphene nanowalls (GNWs) on Cu through PECVD has been increasingly studied, yet the underlying mechanisms remain unclear. In this study, we examined the GNWs/Cu interface and investigated the evolution of their microstructure using advanced Scanning transmission electron microscopy and Electron Energy Loss Spectroscopy (STEM-EELS). GNWs interface and initial root layers of comprise graphitic carbon with horizontal basal graphene (BG) planes that conform well to the catalyst surface. In the vertical section, the walls show a mix of graphitic and turbostratic carbon, while the latter becomes more noticeable close to the top edges of the GMWs film. Importantly, we identified growth process began with catalysis at Cu interface forming BG, followed by defect induction and bending at ‘coalescence points’ of neighboring BG, which act as nucleation sites for vertical growth. We reported that although classical thermal CVD mechanism initially dominates, growth of graphene later deviates a few nanometers from the interface to form GNWs. Nascent walls are no longer subjected to the catalytic action of Cu, and their development is dominated by the stitching of charged carbon species originating in the plasma with basal plane edges.

## Introduction

Vertically aligned few-layer graphene sheets, known as graphene nanowalls (GNWs), exhibit a resemblance to vertically aligned carbon nanotubes when adhered to a supporting surface^1–5^. GNWs possess numerous remarkable physical properties inherent to graphene, such as high electrical and thermal conductivities within the plane. Additionally, the unique geometric arrangement of GNWs results in a significantly higher density of exposed edges per unit area compared to the "flat-on-substrate" graphene films created through thermal chemical vapor deposition (CVD). These distinctive characteristics of GNWs make them well-suited for a wide range of applications in diverse devices, including energy storage systems, gas sensors, biosensors, and heat dissipation devices^2–30^.

The production of GNWs films presents substantial challenges, and a comprehensive understanding of the underlying growth mechanism is essential for addressing them effectively. In general, GNWs are fabricated using energy-intensive synthesis processes such as plasma-enhanced chemical vapor deposition (PECVD)^2,12–26,31–36^. While the substrate plays a crucial role, other PECVD growth parameters, such as plasma characteristics, reaction temperature, and gas flow conditions, are highly diverse and critical. Various semiconducting and insulating materials, including Si, Ge, SiC, SiO_2_, MgO, MnO_2_, and quartz, have been used as substrates in PECVD^12–26,31–34,37^. Due to difficulties associated with the growth process, a preferred strategy involves using transition metals that are catalytically active toward the decomposition of hydrocarbon gases (e.g., Ni and Cu). Interestingly, Cu helps to homogenize the morphology of the walls and ensures height uniformity for the growth of wafer-scale GNWs films^12–14,34,37–41^.

The nucleation and growth of GNWs during the PECVD process are important research topics. Regarding the investigation of the nucleation of vertical structures in carbon onions, buffer layers, or any other surface type, feasible explanations include classical thermal CVD concepts such as the moving boundary model and surface diffusion^26,31,42,43^. Generally in CVD fabricate by plasma hydrocarbon precursor molecules experience inelastic collisions with charged species, thereby developing free radicals, ions, and other reactive species in the reactor chamber. Electric fields build up close to the substrate surface may promote directionality by aligning reactive species with the exposed edges of the vertical graphene. Additionally these electric fields can locally differ because of the presence of lattice strain and defects, surface roughness/impurities, or even thermally induced surface rearrangements (i.e., grain orientation) can influence GNWs formation. With these many interdependent parameter to governs the final critically sophisticate fabrication of GNWs, it is important to understand the nucleation and growth of GNWs at the C/metal interface^4,44–46^, because lack key microstructural understanding remains concerning the C/metal lattice relation and the type of carbon originating and evolving from the interface.

Analytical instrumentation such as optical emission and photoelectron detectors can be integrated with the chambers of PECVD reactors, thus providing real-time information on the GNWs growth process. Furthermore, through their combination with postmortem analysis techniques such as X-ray diffraction (XRD), electron microscopy, and Raman spectroscopy, it is possible to obtain a powerful set of data that characterize these films^47–49^. To identify phenomena at the interface and understand the nature of the wall root, a more localized view is required, ideally at the nanoscale. For example, while Raman spectroscopy can identify the type of carbon (e.g., amorphous, turbostratic, and graphitic), which is limited by the micrometer-sized probe and shape/structure variations that occur along/across the height of walls. Hence, it is required to observe the C/metal interface using techniques that can resolve not only GNW lattice alignment changes but also the nature of C–C bonds (hybridization).

Here, we provide an in-depth investigation of the growth mechanism of GNWs through transmission electron microscopy (TEM). Well optimize wafer-scale patterned GNWs films were grown on square centimeter Cu wire mesh substrates using low-power direct-current PECVD, achieving wall heights of ~ 300 nm, as reported in our previous work^50^. We performed TEM-based advanced techniques, such as the parallel beam mode high-resolution (HRTEM), and convergent beam scanning TEM (STEM) mode combined with High-Angle Annular Dark-Field Imaging (HAADF-STEM) and Electron Energy Loss Spectroscopy (STEM-EELS) mapping of plan-view and cross-sectional (C/Cu interface) GNWs films. These techniques enabled us to assess the growth formation, graphitization degree of the GNWs and its variation along and across them at an atomic scale.

## Results and discussion

Well-optimized, wafer-scale patterned graphene nanowire (GNW) films, grown on square centimeter copper wire mesh, were selected for this study^50^. High crystallinity of the film is confirmed by Raman spectroscopy, and detailed spectra are provided in supplementary figure (Figure S1). SEM images further confirm the growth (Figure S2). The tunability of properties and applications of graphene nanowires (GNWs) is deeply rooted in a detailed understanding of their growth mechanisms and nanostructural characterization. To achieve a comprehensive understanding of these aspects, we employed a combination of imaging and spectroscopy techniques. Our approach included the examination of planar view samples, which were prepared using the lift-off method after chemically etching to remove the copper substrate. Additionally, we prepared cross-sectional samples using focused ion beam (FIB) techniques. By analyzing these samples from both cross-sectional and plan views, our goal was to gain deeper insights into how the growth orientation of GNWs is dependent on the substrate. This multi-faceted approach allowed us to thoroughly investigate the intricate details of GNW growth and orientation, contributing to the broader understanding of their tunable properties and potential applications.

### Plane view microstructural analysis

#### Plane view study by HRTEM

**Figure 1:**
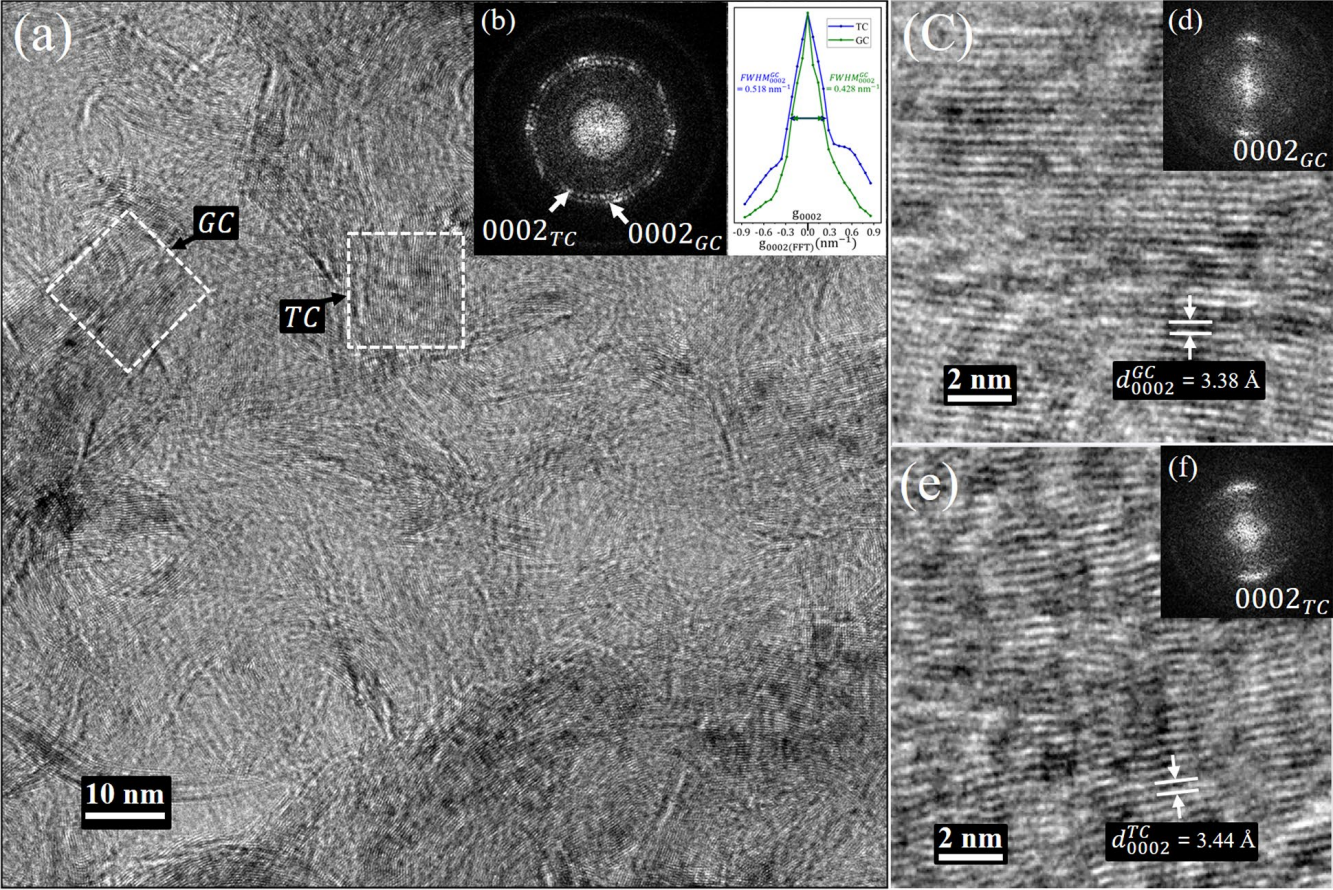
(**a**) HRTEM of GNWs on a Cu mesh and (**b**) corresponding FFT demonstrating two rings of ${(0002) }_{GC}$ and ${(0002) }_{TC}$ that belong to graphitic carbon (GC) and turbostratic carbon (TC), and g0002 profile of both GC and TC from FFT showing TC has broader arc width (FWHM) than GC. The HRTEM and corresponding FFT images of (**c**, **d**) GC and (**e**, **f**) TC are shown. The interplanar distances of GC and TC are ${d}_{0002}^{GC}$= 3.38 Å and ${d}_{0002}^{TC}$ = 3.44 Å, respectively.

The plane-view TEM characterization of GNWs provides an understanding of the top-view projection of the growth structure such as wall gaps, wall assembly, and morphology. Figure 1 shows the plane view HRTEM of GNWs, where two types of graphene wall structure (corresponding to two rings) can be identified from the fast Fourier transform (FFT) (Fig. 1b). These rings with different diameters correspond to different ${d}_{0002}$ (d-spacing of $(0002)$ graphene), which belong to graphitic carbon (GC) and turbostratic carbon (TC)^51^, and the appearance of the ${d}_{0002}$ spot as a ring is attributed to the distribution of GNWs in all directions, and due buckling of same of GNWs at top edge. GC and TC domains are marked with the dotted square in Fig. 1a. The HRTEM and FFT images of GC and TC are shown in Fig. 1c,d and Fig. 1e,f Respectively. Figure 1c,d shows the HRTEM of GC are well-graphitized, confirmed by the narrow ${d}_{0002}$ spot, suggesting reduced lattice strain in GC. Compare to GC, TC has additional defects, evident from the broad ${d}_{0002}$ peak in the FFT and confirm by FWHM profiles of FFT bands (inset Fig. 1b) were TC has a broader ${d}_{0002}$ arc than GC. TC is discontinuous and exhibit higher ${d}_{0002}^{TC}$ (3.44 to 3.52 Å) compare to continuous GC (${d}_{0002}^{GC}$= 3.38 Å). These defects potentially form 3D ${sp}^{3}$ hybridization in graphene. A later section will detail how defects and strains facilitate the upward bending of graphene and GNW formation during growth.${d}_{0002}$ varies from C/Cu interface (BG) to vicinity of top edges of the GNWs to the C/Cu interface (BG) and have strong correlation with graphitization as discussed later.

#### Plane-view spectroscopy analysis by EELS

**Figure 2:**
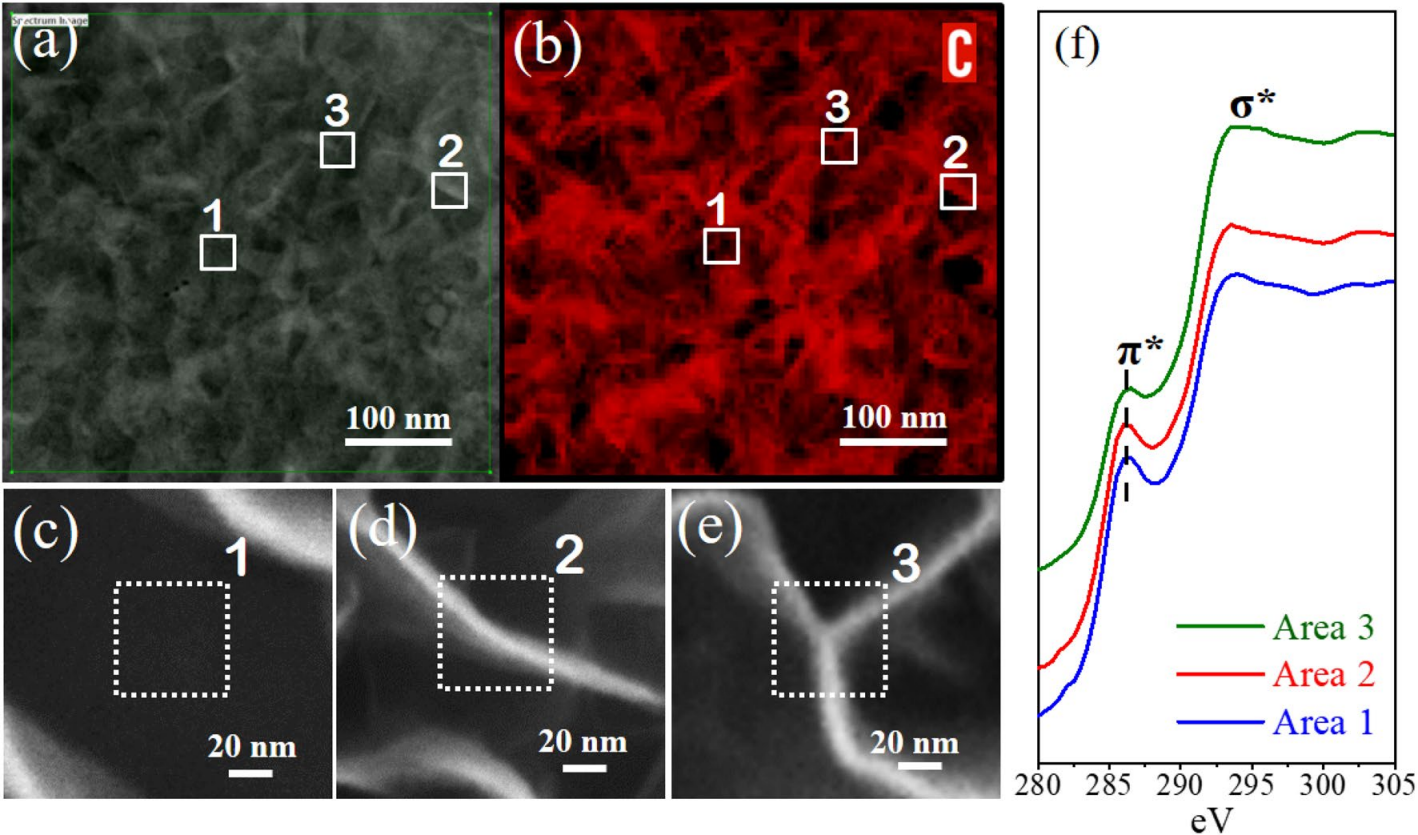
(**a**) Plane-view HAADF-STEM image and (**b**) corresponding Carbon K-edge EELS elemental map (red) with the areas of interest marked as 1, 2, and 3. (**c**–**e**) SEM images collected from Areas 1, 2, and 3. (**f**) Carbon K-edge core-loss EELS spectra of the respective areas.

In Fig. 2, the plan view of GNWs is presented by HAADF-STEM-EELS methods. These techniques facilitate the investigation of the GNWs' morphology, distribution, and the degree of graphitization within their walls. Figure 2a displays the HAADF-STEM image of GNWs' plan view, along with STEM-EELS C K-edge mapping (Fig. 2b) from the green square-highlighted region. This C K-edge map is instrumental in visualizing the distribution of the GNWs, offering a detailed view of their spatial arrangement and structural features. To do so , three specific areas of interest selected for an in-depth investigation into graphitization, as indicated by white solid square regions marked as 1,2,3 (Fig. 2a,b) and magnified SEM images of these are given in Fig. 2c–e. Area 1 represents the gap between GNW walls, providing access exclusively to the bulk graphene (BG) underneath. Area 2 features a single, independent GNW root, allowing access to the BG beneath it, and Area 3 showcases a collection of interconnected GNWs, including merged GNWs, their merging points, roots, and the BG beneath them.

The normalized C-K core-loss EELS spectra corresponding to areas 1–3 from HAADF and EELS mapping shown in Fig. 2f. The C-K core-loss EELS spectra consist of the π* (283 eV) and σ* (293 eV) edges, which correspond to graphitic (2D) planar ${sp}^{2}$ hybridization and 3D diamond-like ${sp}^{3}$ hybridization, respectively. GC exhibits a dominant π* (${sp}^{2}$) edge compared to TC, confirming its higher level of graphitization. This is attributed to the fact that TC has more planar defects than GC.${sp}^{3}$ contribution increases because of the presence of defects and the interaction of dangling C bonds with the adjacent graphene layer to create 3D bonds in graphene-like structures and its observed here in TC. ${sp}^{2}$ is dominant for area 1, and its impact reduces till area 3. In Area 1, strong ${sp}^{2}$ dominates due to graphitic nature for BG. In Area 2, where a single GNWs is present along with BG, the presence of defects near the root and TC near the GNW's edge result in a reduction in ${sp}^{2}$ hybridization (or relative increase in ${sp}^{3}$). Additionally in area 3, additional 3D ${sp}^{3}$ hybridization added by multiple GNWs merging points. Area 3 exhibit an additional reduction in ${sp}^{2}$ (π*) hybridization, suggesting that defects and TC dominate in merged GNWs.

### Cross-sectional microstructural analysis

The cross-sectional lamella of a GNWs film along with Cu was prepared for interfacial characterization. The collective crystallographic understanding of both vertical and basal parts with Cu was examined by electron diffraction and HRTEM.

#### Selected-area electron diffraction (SAED)

**Figure 3:**
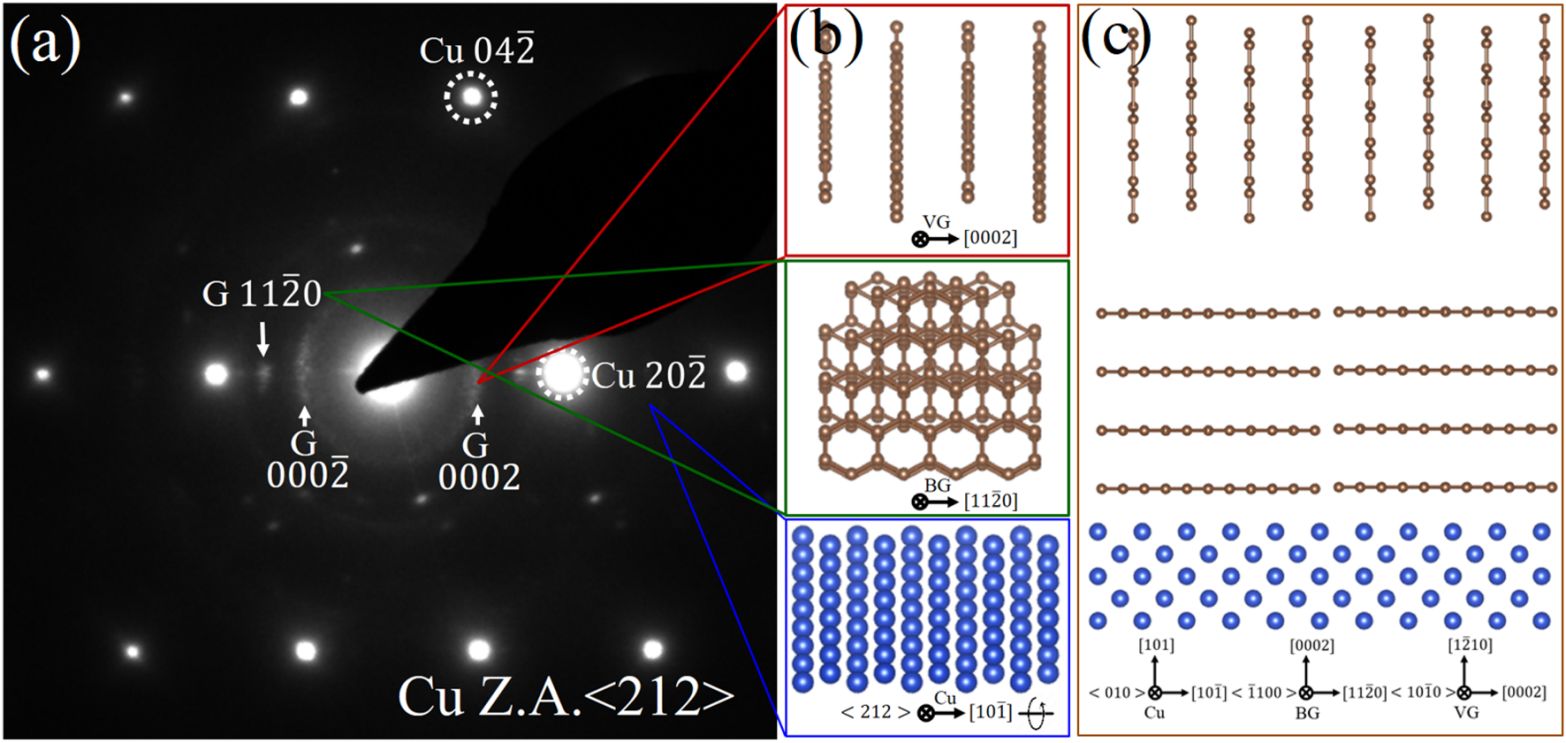
(**a**) Selected-area electron diffraction pattern (SAED) of the GNWs/Cu interface along Cu Z.A. $<212>$, which is then (**b**) Reconstructed in real space and shows the morphologies of the basal and vertical parts of GNWs. (**c**) Further reconstructed of combined crystal orientation by rotating 70.2° along Cu $2\times (10\overline{1 }$) axis to preferable orientation to represent (0002) both VG and BG.

The Fig. 3a show the SAED pattern of the GNWs/Cu interface, aimed at gaining insights into the growth morphology. The pattern clearly reveals the presence of two distinct graphene morphologies along the Cu $\left[20\overline{2 }\right]$ direction, represented by the two graphene ED spots: $(0002)$ for VG and $(11\overline{2 }0)$ for BG. This SAED pattern confirms the bulk crystallinity of graphene in the GNWs film. Figure 3b. Shows the reconstruction of graphene and Cu based on SAED, providing a 3D perspective of the development of graphene morphology, similar information can be obtained through tilting experiments in the TEM^52^. It offers a projection along the Cu zone axis $<212>$ and direction $2\times (10\overline{1 }$) along with BG $(11\overline{2 }0)$ and GNWs $(0002)$. Additionally, Fig. 3c demonstrates the structure's reconstruction through rotation along Cu $(10\overline{1 }) ,$ providing a clearer view of BG and VG of GNWs. The arc $\left(0002\right)$ of graphene (Fig. 3a) highlights that different sets of GNWs exhibit curling along vertical distribution.

#### Cross-sectional analysis by HRTEM

**Figure 4:**
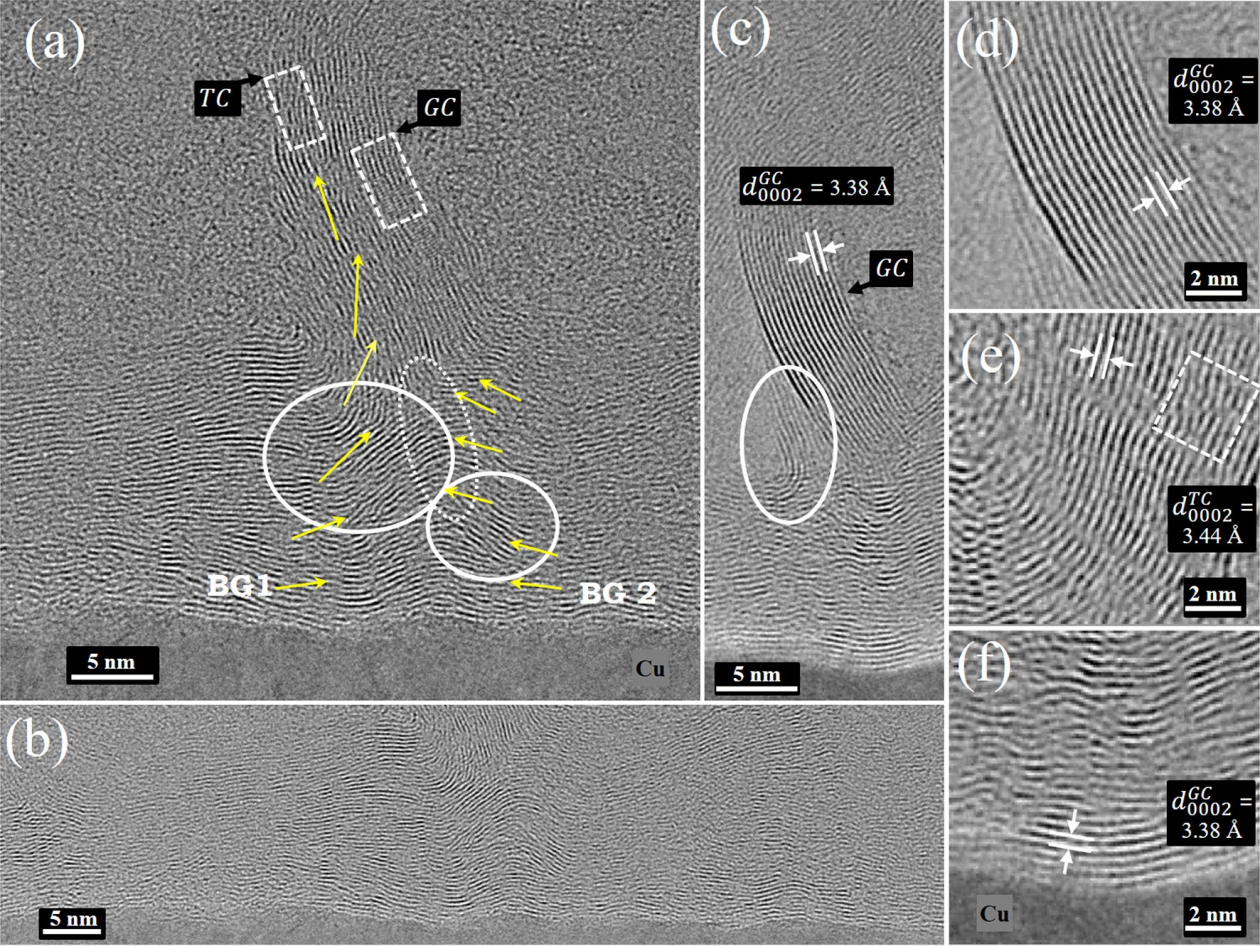
HRTEM of GNWs near the interface showing (**a**) coalescence of two basal graphene (BG) and formation of a GNWs. (**b**) structural morphology of basal graphene (BG) following the miniature roughness of the Cu substrate. (**c**) disconnected GNWs on BG. (**d**) graphitic carbon (GC). (**e**) turbostratic carbon (TC) with defects in the vertical part of graphene. TC shows bending and planar defects (dotted circle). (**f**) initial layers of BG as pristine GC form by catalysis.

The coexistence of two carbon types in GNWs necessitates an investigation into their synthesis dynamics and intrinsic properties, emphasizing the critical roles of catalytic processes of hydrocarbon-Cu involvement during formation in the PECVD chamber. Given the technical constraints that preclude in situ TEM within the PECVD apparatus, our methodology pivots to a rigorous post-synthesis examination. To achieve this, it is essential to analyze the structure from the Cu substrate interface to the top edge of the GNWs, which can be effectively examined using Cross-sectional TEM. HRTEM image demonstrate the formation of GNWs from Cu interface BG, VG regions, and the few-layered edge section of VG, as illustrated in Fig. 4. Figure 4a shows the uniform formation of BG near the substrate. HRTEM shows that graphene (BG) continuously forms from the interface, suggesting that the hydrocarbon-Cu catalysts forms initial layers of carbons crystallize from the very substrate and are not deposited as amorphous carbon. The HRTEM show that the initial layers of BG are continuous and defect-free up to ~ 3 nm (~ 10 BG layers), as shown in Fig. 4b,f. These BG layers dominantly involve GC. After 10–15 layers from the Cu interface, BG layers meet with other neighboring layers and start mismatching, forming defects, and vertically bending. The bending of BG and planar dislocations are marked with the white circles and arrows in Fig. 4a. Defects and bending have local effects on ${sp}^{2}$ hybridization in graphene because they lead to 3D ${sp}^{3}$ hybridization. Rigorous analysis of the role of hybridizations using EELS later in article.

The Fig. 4b shows the HRTEM of the continuous BG part. The basal part acts as a support for the formation of GNWs and is important for the morphology development of GNWs. The thickness of BG determines the flexibility of GNWs and has applications in stretchable devices^53^. Plasma exposure increases the temperature of the substrate and thus can cause the formation of local defects and create a potential gradient close to the interface. Consequently, these irregular electric fields on the surface can effectively increase the diffusion coefficient and changes the morphology. The structural morphology of BG follows the miniature roughness of the Cu substrate, and this roughness sometimes bends the following graphene layers, resulting in dangling graphene and defects during growth and then leading to vertical formation. Other possible factors such as the role of roughness and charge accumulation in Cu (dark contrast areas in the circle in Figure S3) for vertical formation are important. Presumably, this dark contrast in Cu is attributed to local charge accumulation, which can be attributed to local strain that causes different electromagnetic (EM) fields close to strained areas. These local changes in the EM field affect hydrocarbon plasma, which acts differently compared to the surrounding during the growth. Hence, different growth morphologies occur close to these charge-accumulated dark contrast areas. This charge-accumulated dark area can occur in the Cu substrate interface because of local strain, defects, a local grain boundary in Cu, the deformation of Cu at high temperatures, and a defect dent created by plasma in the CVD chamber. These multiple factors might play an important role in the growth of the local morphology of GNWs. The controlled growth at the interface can then tune the final morphology of GNWs.

The Fig. 4c show HRTEM of GNWs on BG, which appear disconnected from the BG. These GNWs exhibit continuity from different roots of the BG (BG1&2), as highlighted in Fig. 4a,c. The continuation and bending of these GNWs from various neighboring BG are marked. GC and TC in the vertical part of the GNWs are shown in Fig. 4d,e. The presence of GC in GNWs (Fig. 4d) confirms the occurrence of graphitization within the GNWs, enhancing the intrinsic properties of pristine multilayer graphene. ^54^. Whereas TC part of GNWs does not possess pristine graphene properties. The comparative properties of these two phases can provide the final properties of GNWs for device applications. Figure 4f shows the initial layers of pristine graphene on Cu. The initial layers of BG form at the interface where catalysis between hydrocarbon plasma and Cu occurs. This results in the initial pristine deposition of BG as domains over the Cu substrate area. Furthermore, these domains grow and meet at coalescence points. In these intersection points of BG domains, BG starts to vertically bend, as shown in Fig. 4a. These coalescence points act as nucleation points for the growth of VG. These bent graphene at the coalescence point have dangling bonds, where further vertical growth occurs. A similar observation finding was reported in vertical 2D ${ReS}_{2}$ on dangling ${MoS}_{2}$ bonds ^55^. The formation of GNWs at the coalescence points of two horizontal BG domains is discussed in detail in Section "Cross-sectional spectroscopic analysis using EELS" based on both HRTEM and EELS results.

#### Cross-sectional spectroscopic analysis using EELS

**Figure 5:**
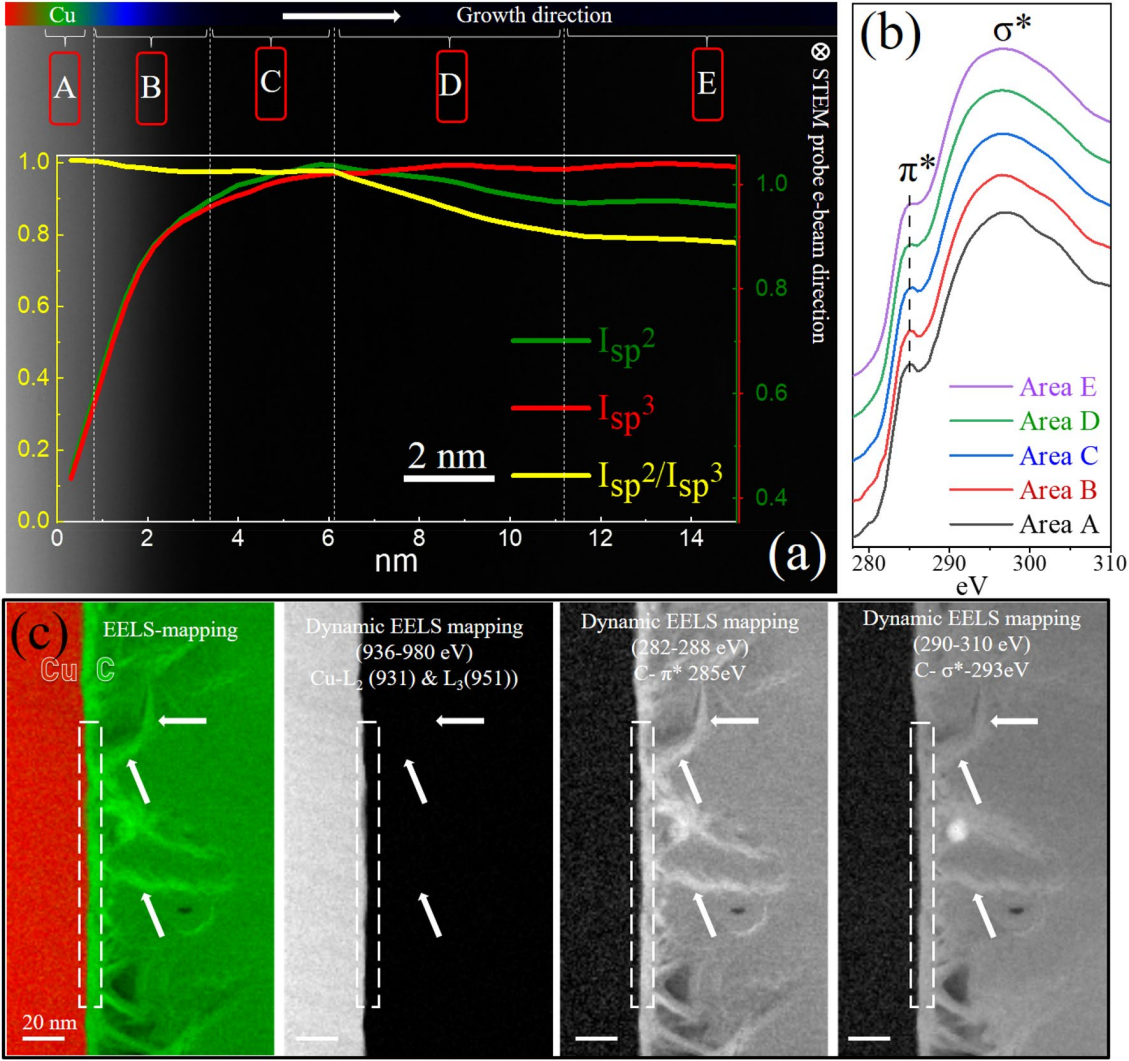
(**a**) STEM-HAADF image of PECVD grown GNWs on Cu from the beginning of interface toward the growth direction (left to right). The embedded graph shows the normalized contribution of ${sp}^{2}$(π*) and ${sp}^{3}$(σ*) and the ${{\text{I}}sp}^{2}/{{\text{I}}sp}^{3}$ ratio in the dedicated areas (A–E) along the growth direction, suggesting a change in carbon hybridization/graphitization. Areas A, B, C, D, and E are marked as the bases of the ${sp}^{2}/{sp}^{3}$ contribution of carbon. (**b**) Corresponding carbon K-edge core-loss EELS spectra of areas A–E. (**c**) EELS mapping of GNWs on Cu, along with dynamic EELS mapping of Cu (L_2_&L_3_), π*, and σ*, reveals significant features. Strong π* EELS signals from both the interface (dotted square) and the side edge (arrow) of GNWs confirm the dominant graphitic hybridization.

Cross-sectional STEM-EELS characterization was performed for an in-depth understanding of the steps of GNW growth along the growth direction with complete thickness information about BG, GC, and TC. Figure 5. shows the growth of the GNWs film in growth direction. The HAADF image with the graph of the normalized contribution of ${sp}^{2}$(π*) and ${sp}^{3}$(σ*) and the ratio (${{\text{I}}sp}^{2}/{{\text{I}}sp}^{3}$) of carbon K-edge core-loss EELS along the growth direction are shown. The graph is generated by the EELS elemental mapping of the overall areas from the Cu interface, BG, and the vertical part of GNWs. STEM-EELS elemental mapping with a nanoprobe was performed using an advance Cs corrected microscope with monochromator and EELS detector because e-beam is an extremely precise and beam-sensitive technique, particular for graphene. The graph is registered with the HAADF dimension along the growth direction. The normalized intensities ${{\text{I}}sp}^{2}$ and ${{\text{I}}sp}^{3}$ were obtained from the ${sp}^{2}$ (285 eV) and ${sp}^{3}$(293 eV) EELS elemental mapping from the areas in the HAADF-STEM image. Both ${{\text{I}}sp}^{2}$ and ${{\text{I}}sp}^{3}$ together suggest the 2D (graphitic) and 3D (turbostratic) nature of graphene, respectively, and the level/degree of graphitization in the related areas.

The growth evolution of GNWs is divided into five-part area segments A–E based on EELS elemental mapping and spectra, as well as HRTEM characterization. Figure 5b shows carbon K-edge core-loss EELS spectra from areas A–E. EELS data is generated from the e-beam transmission through the total thickness in the e-beam direction and involves all the information of the marked areas. For example, area A (Fig. 5a) and corresponding spectra (Fig. 5b) represent the overall thickness information of marked area A along the e-beam direction. Area A is the Cu interface where the C K-edge EELS signal starts and is the primary location where the initial Cu and hydrocarbon catalysis and the start of the formation of a graphene-like structure occur. This is supported by the EELS mapping spectra of A where ${{\text{I}}sp}^{2}$ and ${{\text{I}}sp}^{3}$ start appearing as graphene forms from the interface (Fig. 5a). In area B where the first step of catalysis occurs, well-aligned BG starts forming. This is the start of the nucleation of the few primary layers of pristine BG. HRTEM images show the BG formation at the interface (Fig. 2b–e), confirmed by EELS because ${sp}^{2}$ contribution dominated in area B compared to top areas. In area B, the highest form of well-aligned graphene forms as can be observed from ${{\text{I}}sp}^{2}$ and ${{\text{I}}sp}^{3}$ increase while ${{\text{I}}sp}^{2}/{{\text{I}}sp}^{3}$ ratio nears ~ 1.02. This suggests the domination of the pure graphitic nature. In area C (~ 3 nm away from the interface) small contributions of initial defects start to form in the graphitic structure, and these defects contribute to the formation of 3D ${sp}^{3}$ hybridization. It appears as a slight decrease in ${{\text{I}}sp}^{2}$ and ${{\text{I}}sp}^{2}/{{\text{I}}sp}^{3}$ along the growth direction but remains graphitic as ${sp}^{2}$ peaks are prominent in the EELS spectra (Fig. 3b, Area C). Therefore, areas C are the nucleation (root) area that lay down the foundation of the vertical growth of graphene. Moreover, HRTEM shows area C where the root (of nucleation) of GNWs starts forming (Fig. 4a, dotted circle). In area D, the rapid change in the ${{\text{I}}sp}^{2}/{{\text{I}}sp}^{3}$ ratio is observed because of the increase in defects, which initiates basal graphitic carbon bending toward the vertical direction and GNWs formation (Fig. 4a,b, marked as solid circles). The core-loss EELS ${sp}^{2}$ peak for area D significantly reduces, confirming this result (Fig. 5b). An important appearance of TC can be observed in area D (HRTEM). The rapid change in ${{\text{I}}sp}^{2}/{{\text{I}}sp}^{3}$ ratio saturates in area E, which ends at the tip of GNWs. This is the bulk formation of GNWs, which comprise both GC and TC. This confirms that the overall ratio of the distribution of both GC and TC remains the same throughout area E. Microstructural formation of GNWs on Cu (catalyst) from interface to bulk further confirm by dynamic EELS mapping of Cu (L_2_&L_3_), π*, and σ* ((Fig. 5c). Strong π* EELS signals from both the interface (dotted square) and the side edge (arrow) of GNWs confirm the dominant 2D graphitic hybridization.

In summary, Area B is where the catalytic nucleation and formation of BG occur, while area C involves the initiation of a few defects and the bending of BG. These defects and bending become prominent in area D, leading to the initial formation of the root of GNWs. In area D, GC is present and TC starts to form. Starting from area E and onwards, the formation of both TC and GC saturates, leading to the bulk formation of both TC and GC.

### Growth evolution of GNWs

**Figure 6:**
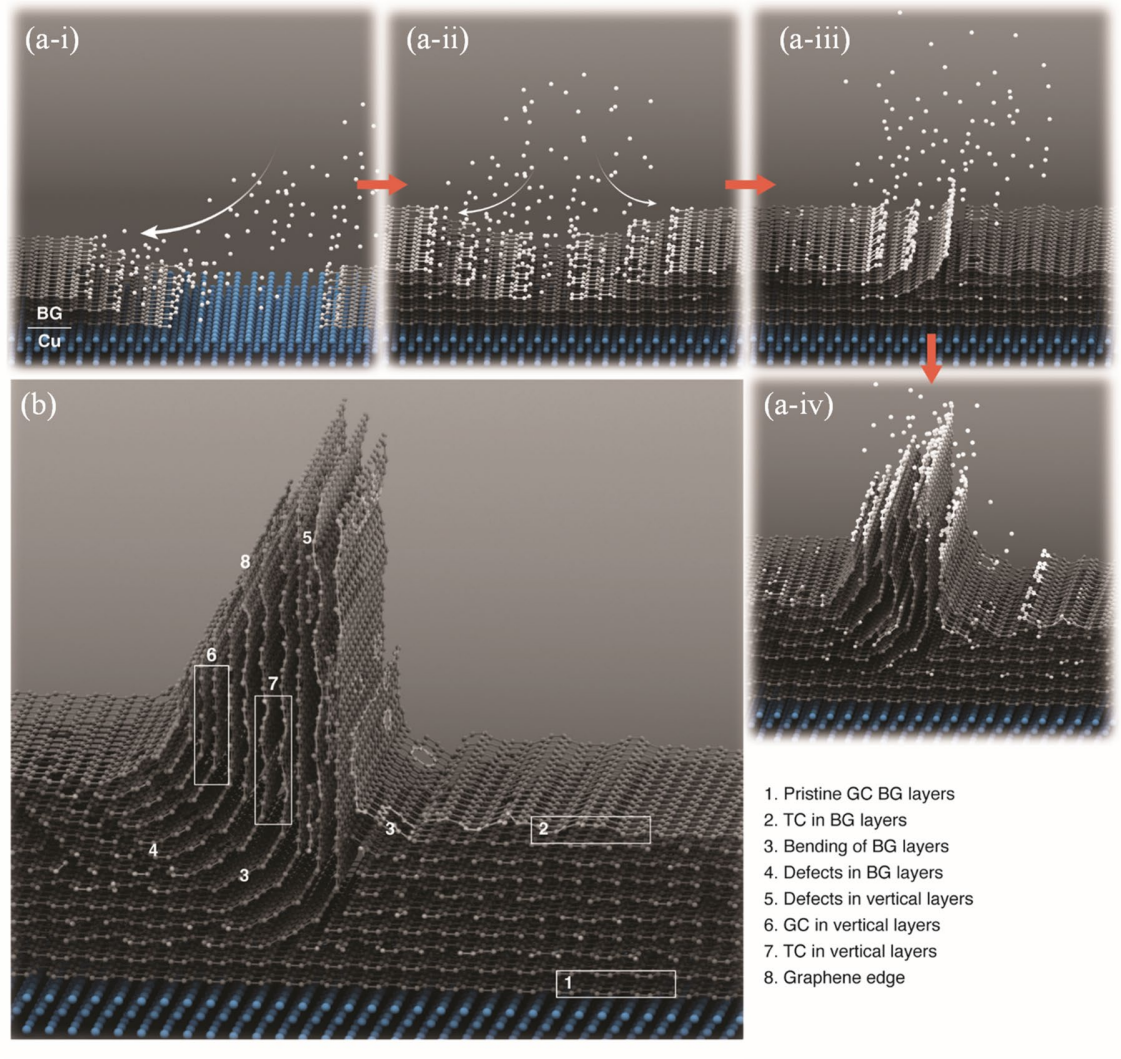
Schematic of (**a**) growth evolution (i–iv) of GNWs from the Cu substrate interface. (i) Initial formation of two domains of pristine BG on top of the Cu substrate, (ii) Coalescence of these BGs during formation, (iii) Vertical bending of one BG domain, forming the root of GNWs, (iv) Further deposition of C and the formation of vertical GNWs. (**b**) Microstructural details of growth evolution in GNWs on Cu.

The growth evolution of GNWs is shown in Fig. 6. An illustration is obtained by compiling informations from SAED (Fig. 3),  HRTEM of the two BG domains that meet to form vertical GNWs (Fig. 4a), and dynamical STEM-EELS mapping (Fig. 2, and Fig. 5) . The initial layers of the horizontal BG domain form at interface catalysis between hydrocarbon plasma and Cu (Fig. 6a–i). The initial few layers of BG domains are pristine well-graphitized graphene, as confirmed by HRTEM (Fig. 4a,b,f) and EELS of area B in Fig. 5. Figure 6a–i shows the coalescence of these two BG domains with incoming hydrocarbon/plasma. Here, the domain boundary meets and potentially forms mismatch defects, which locally disturb ${sp}^{2}$ hybridization (this microstructure can be seen by HRTEM, shown in Fig. 4a, and in area C with slight decreases of ${{\text{I}}sp}^{2}/{{\text{I}}sp}^{3}$ (Fig. 5)). Note that additional growth at these coalescence points leads to the vertical bending of one domain and forming the root or nucleation area for VG or GNWs, as shown in Fig. 6a(iii). These are the same areas where the presence of mismatch, defects, and bending becomes prominent and ${sp}^{2}$ decreases, which sometimes potentially leads to the formation of TC at some of these root points, where ${{\text{I}}sp}^{2}/{{\text{I}}sp}^{3}$ further decrease, as shown in areas C and D in Fig. 5. As both GC and TC are reported in the vertical part of GNWs, the decrease in ${sp}^{2}$ and defects, bending, and TC start appearing ~ 2–4 nm from the Cu interface. Finally, the GNWs vertically reshape with the incoming plasma-dissociated carbon precursor, as shown in Fig. 6a(iv). The initial basal part of GNWs is formed by catalytic action at the Cu interface within a few nanometers, and then the vertical part is no longer subjected to the catalytic action of Cu, and their development becomes dominated by the stitching of charged carbon species that originate in the plasma.

The Fig. 6b shows the final GNWs illustration based on the TEM study, enriched with microstructural details. It illustrates all the microstructural specifications observed from the Cu interface to the edge of VG such as the few layers of pristine BG formed at the Cu interface because of the catalytic reaction between Cu and hydrocarbon plasma. TC in BG is reported away from the interface because of defects, leading to the bending of BG at coalescence points and is responsible for the vertical formation, the appearance of planar defects as extra graphene layers between vertical layers, GC in vertical layers, TC in vertical layers, and dangling graphene edges at the top of GNWs.

## Conclusions

Herein, the morphology and structure of the GNWs grown on a catalytic Cu substrate have been investigated. In particular, we imaged and analysed the formation and growth of GNWs. Consistent results could be achieved using different datasets (HRTEM, SAED, and STEM-EELS elemental mapping) provided consistent results, allowing us to identify the formation of well-crystalized GC with a thickness of a few manometers on the surface of the Cu substrate. From this region, the basal planes bend upward and the walls vertically grow. Importantly, the growth direction changes because of the coalescence of different surface grains. When neighboring grains started to merge, they start forming defects due to mismatch, and these defects and initial bending and forming dangling bands pointing upwards, these dangling bonds act as nucleation points for the formation of vertical part of GNWs. The lack of the directing effect of the Cu surface leads to the coexistence of GC and TC in the walls, influencing much of the GNWs film properties. We believe that the localized structural evolution information may be useful to understand how these films can be further utilized in applications ranging from battery electrodes, flexible/stretchable electrodes to wearable electronics.

## Methods

(i) Growth of GNWs.

The procedure for GNWs growth was recently reported by our group^50^. Briefly, the catalytic substrate comprises a 10 mm × 10 mm square of Cu mesh. The mesh was composed of 0.11 mm-diameter wires that were intertwined to form an array of 0.05 mm × 0.05 mm square-shaped cavities. The Cu substrate was loaded into a commercial PECVD reactor, as shown in Figure S4a (4″ BMPro Aixtron, GmbH, Germany). The system was purged with Ar and evacuated to 10–2 mbar. The mesh was heated to 800 °C with a ramp rate of 75 °C/min in an Ar/H_2_ atmosphere (160/200 sccm), maintaining a chamber pressure of 6 mbar throughout the process. Next, the DC plasma was initiated with a power of 150 W and left to stabilize for 1 min. The carbon feedstock (methane, CH4) was then introduced into the chamber. The Ar/H_2_/CH_4_ plasma was maintained at a constant flow (160/200/60 sccm, respectively) for 20 min with a chamber pressure of 6 mbar and substrate heating at 800 °C. Subsequently, the entire system was cooled down to room temperature at a rate of approximately 40 °C/min using an Ar flow (4000 sccm). The reaction temperature profile and other process parameters are summarized in Figure S4b.

(ii) Characterization techniques.

For transmission electron microscopy (TEM) studies, two types of samples were prepared, i.e., plane-view and cross-sectional. For the plane-view sample, the Cu mesh substrate was eliminated by chemical etching with a solution of FeCl_3_ overnight. The carbon film was left floating on a DI water filled in a Petri dish and then transferred onto a Lacey-carbon Au TEM grid by carefully picking and drying it up (Figure S5). For the cross-sectional sample, a thin lamella of the as-made GNW film, in addition to the Cu substrate, was cut using a focused ion beam (FIB) SEM microscope (FEI Helios G4), equipped with a field emission gun. First, to protect the region of interest (the GNW), three types of protective coatings were deposited on it. Using the e-beam (on coating mode), 0.2-µm and 0.5-µm layers of C and Pt, respectively, protected the top part of the nanowalls. Moreover, a 1.5-µm Pt layer was deposited by ion beam to shield the entire stack. After depositing the protective coatings, the milling procedure was conducted by cutting and thinning down the lamella thickness to 50 nm, making it electron-transparent. High-resolution transmission electron microscopy (HRTEM), high-angle annular dark-field scanning TEM (HAADF-STEM), selected-area electron diffraction (SAED), and electron energy loss spectroscopy (EELS) elemental mapping (in STEM mode) were performed on a double Cs-corrected Thermofisher Titan Themis 60–300 Cubed TEM microscope (operated at 300 kV), equipped with an ultrabright field emission gun (X-FEG) and a Wien-type monochromator. HR-EELS mapping/spectra were obtained employing monochromator, achieving an energy resolution superior to 100 meV. To analyze and process data sets, multiple software packages were employed. The Gatan Digital Micrograph and JEMS suites were used for all image processing and microstructural analyses. Crystalmaker was used to develop the GNW-on-Cu models, and the crystal orientation and diffraction simulations were generated in accordance with the experimental SAED patterns. Digital images were obtained using a commercial smartphone. Scanning electron microscopy (SEM) image was obtained using Nano FEI Magellan (at 1–5 kV, 50 pA). Raman spectroscopy was realized using a spectrometer integrated with a confocal microscope (Alpha300R, WITec) at a laser wavelength of 532 nm using low excitation power (ca. 25%) to avoid heat-induced effects on the nanowalls.

### Supplementary Information

Supplementary Information.

## Data Availability

All relevant data are available from the corresponding authors.
