# Supplementary material for: Graphene nanowalls formation investigated by Electron Energy Loss Spectroscopy
Source: Sci Rep. 2024 Jan 18;14:1658. doi: 10.1038/s41598-023-51106-z (PMC10796779; doi:10.1038/s41598-023-51106-z)
Supplement: Supplementary file 1 — Supplementary Information. [file 41598_2023_51106_MOESM1_ESM.docx]

**SUPPLEMENTARY INFORMATION**

**Microstructural investigation of plasma-enhanced chemical vapor deposition grown graphene nanowalls**

## Authors

Badri Vishal,^a^ * Abdeldjalil Reguig,^a^ Mohammed Bahabri,^a^ and Pedro M. F. J. Costa^a^ *

**Affiliations**

^a^*King Abdullah University of Science and Technology (KAUST), Physical Science and Engineering Division, Thuwal 23955-6900, Saudi Arabia.*


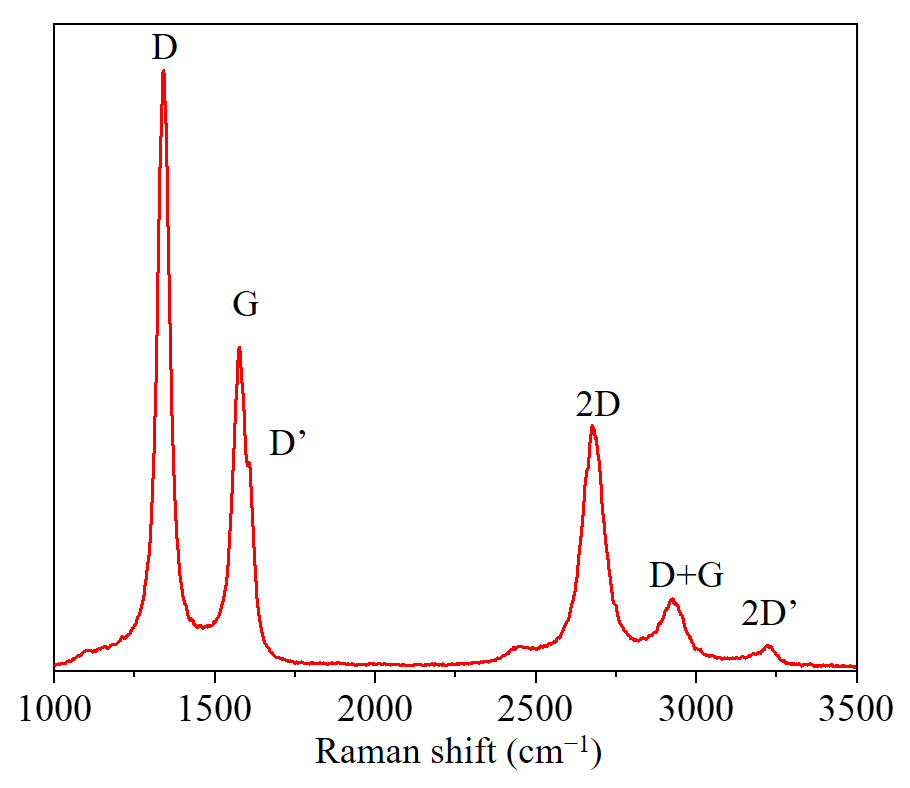


Fig.S1. Normalized Raman spectra of Graphene Nanowalls (GNWs).


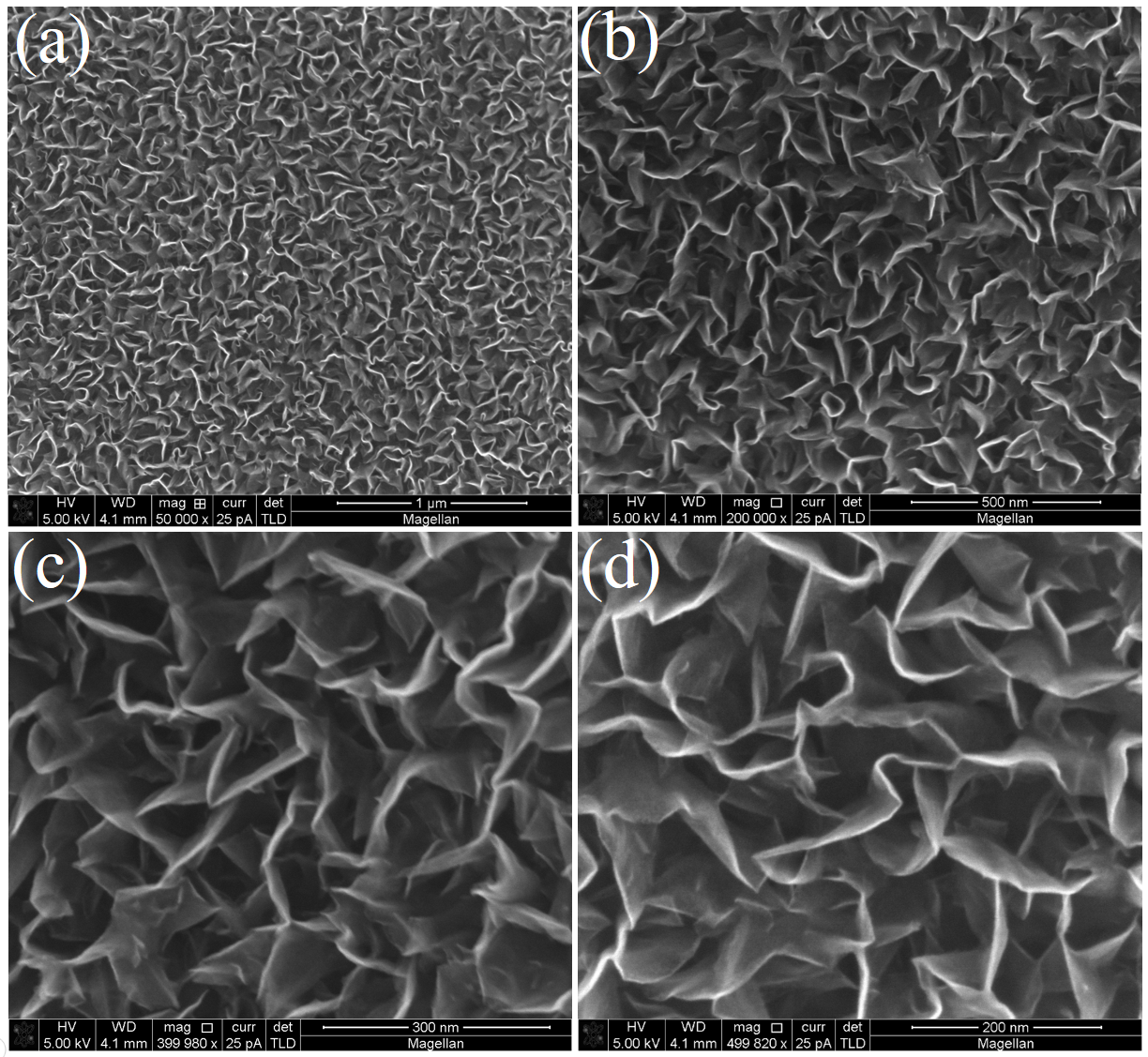


Fig.S2. SEM image of free-standing GNWs with different magnifications.


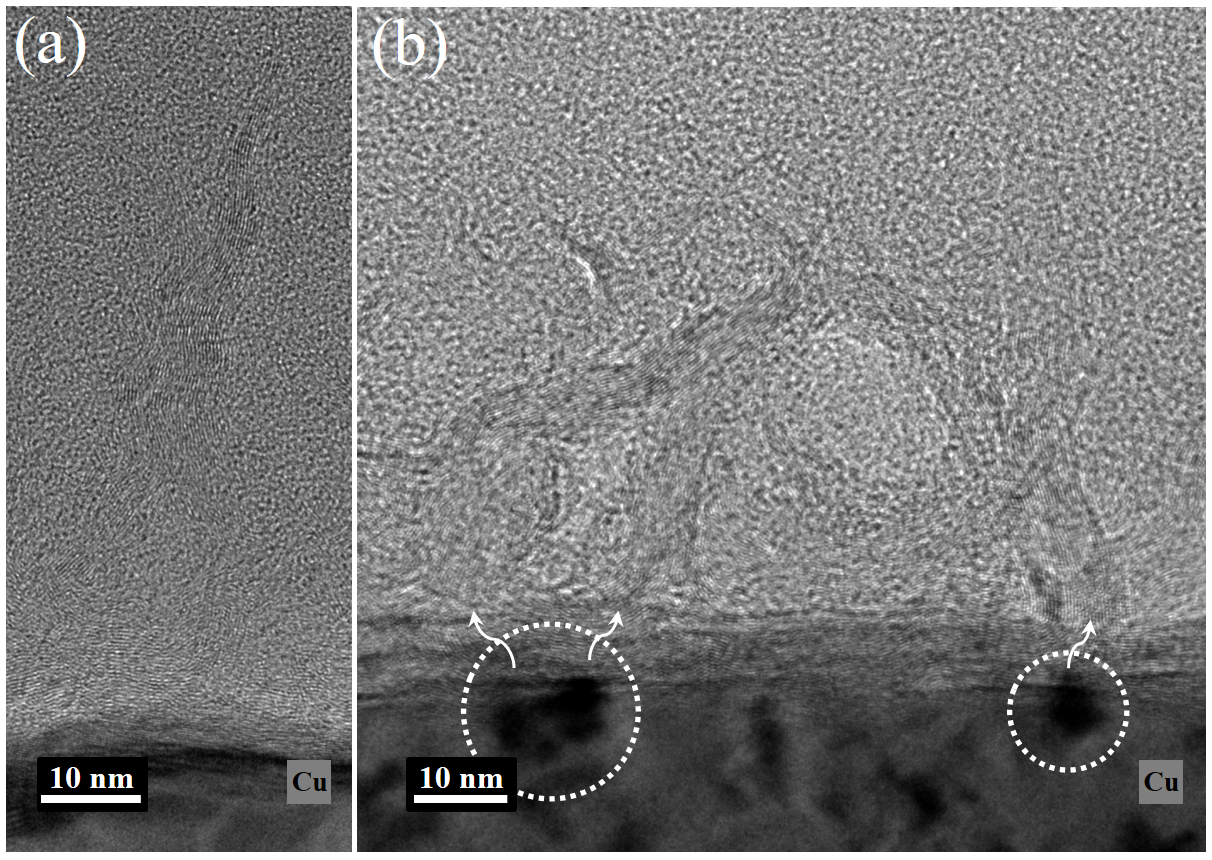
 Fig.S3. Low mag. TEM of GNWs on Cu with the probable role of (a) roughness at the interface leads to vertical growth, and (b) Charge accumulation near Cu interface (at dark contrast mark in a circle). This dark contrast may occur due to more scattering of electron beam during the TEM experiment for the area or can be due to the thickness contrast, strain contrast, or charge accumulation in metallic Cu. Strain contrast is most suitable due to the Cu substrate being heated (till 800°C during deposition). Regardless both local charge accumulation and strain invite change in electric charge and electric field than surrounding, and highly charged hydrocarbon plasma feel in homogeneously locally. This change encourages the deposition to occur differently near these areas.


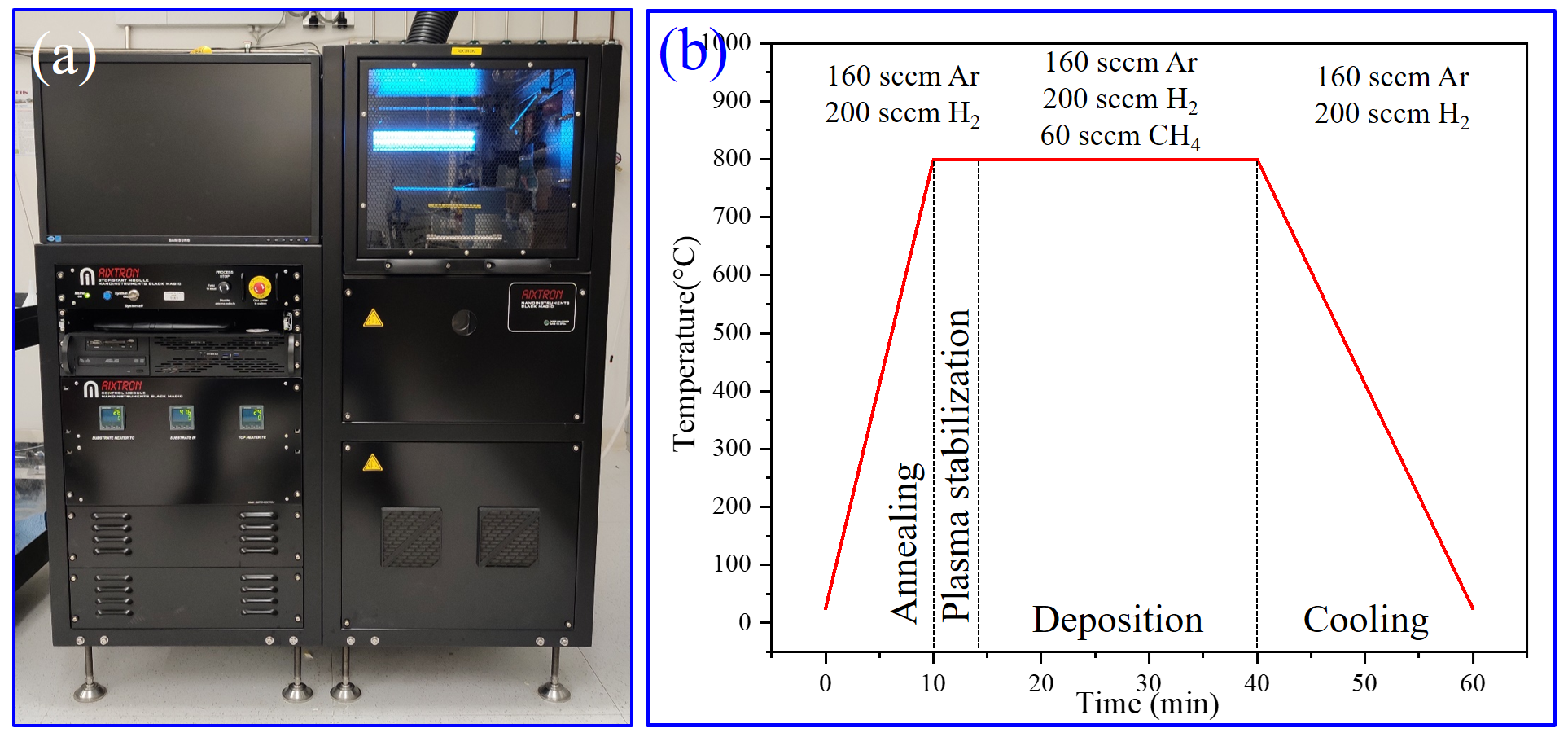
Fig.S4. (a) The commercial PECVD reactor, Aixtron BM Pro 4“. (b) The growth recipe followed to deposit GNW on Cu.


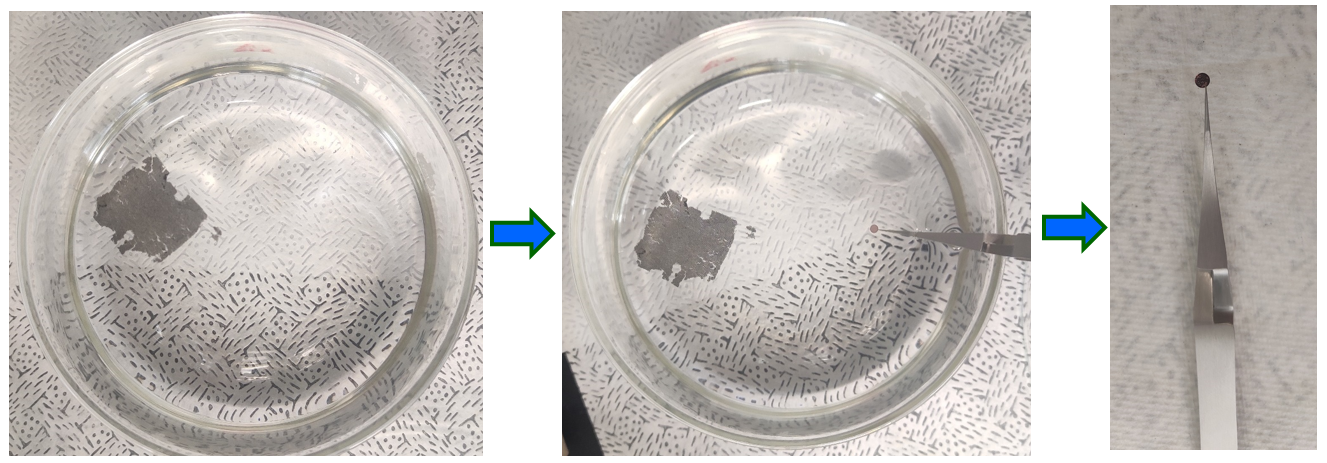


Fig.S5. Steps of plan-view sample preparation (a) Cu substrate is chemically etched by Iron Chloride (FeCl_3_) solution and remaining GNWs are suspended in water **(**b) Suspended GNWs ready to be transferred on lacey carbon-Au TEM grid, **(**c) suspended GNW on lacey carbon-Au TEM grid.


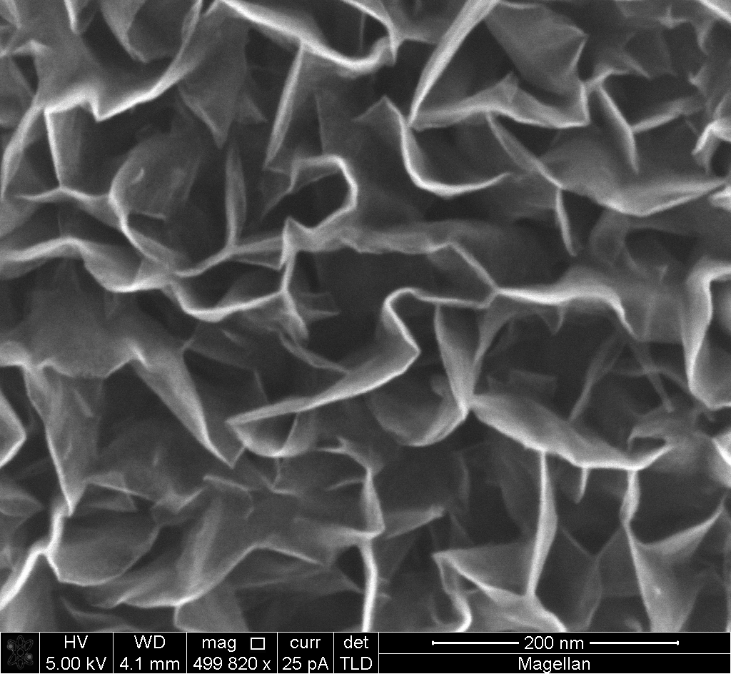


(c)
